# Modelling the temperature suitability for the risk of West Nile Virus establishment in European *Culex pipiens* populations

**DOI:** 10.1111/tbed.14513

**Published:** 2022-03-28

**Authors:** Gabriella Di Pol, Matteo Crotta, Rachel A. Taylor

**Affiliations:** ^1^ Veterinary Epidemiology, Economics and Public Health Group Department of Pathobiology and Population Sciences Royal Veterinary College London UK; ^2^ Department of Epidemiological Sciences Animal and Plant Health Agency Surrey UK

**Keywords:** infectious disease transmission, spatial risk assessment, thermal suitability, vector‐borne, West Nile fever

## Abstract

Increases in temperature and extreme weather events due to global warming can create an environment that is beneficial to mosquito populations, changing and possibly increasing the suitable geographical range for many vector‐borne diseases. West Nile Virus (WNV) is a flavivirus, maintained in a mosquito–avian host cycle that is usually asymptomatic but can cause primarily flu‐like symptoms in human and equid accidental hosts. In rare circumstances, serious disease and death are possible outcomes for both humans and horses. The main European vector of WNV is the *Culex pipiens* mosquito. This study examines the effect of environmental temperature on WNV establishment in Europe via *Culex pipiens* populations through use of a basic reproduction number ($R_{0}$) model. A metric of thermal suitability derived from $R_{0}$ was developed by collating thermal responses of different *Culex pipiens* traits and combining them through use of a next‐generation matrix. WNV establishment was determined to be possible between 14°C and 34.3°C, with the optimal temperature at 23.7°C. The suitability measure was plotted against monthly average temperatures in 2020 and the number of months with high suitability mapped across Europe. The average number of suitable months for each year from 2013 to 2019 was also calculated and validated with reported equine West Nile fever cases from 2013 to 2019. The widespread thermal suitability for WNV establishment highlights the importance of European surveillance for this disease and the need for increased research into mosquito and bird distribution.

## 1 INTRODUCTION

West Nile Virus (WNV) is a mosquito‐borne flavivirus of the Japanese encephalitis virus group (Smithburn et al., 1940) with Lineages 1 and 2 of particular importance due to their association with human disease outbreaks (Petersen et al., 2013). Lineage 1 has been detected globally, including in Europe, in Italy and Spain where it has been endemic since 2010 (May et al., 2011). Until recently, Lineage 2 was mainly found in sub‐Saharan Africa, but after detection in Hungary in 2004 in wild birds (Bakonyi et al., 2006) it has spread rapidly across the south of Europe. It has also moved further north, with numerous cases occurring in Germany and most recently in the Netherlands in 2020 (Vlaskamp et al., 2020). Lineage 2 has replaced Lineage 1 as the most common to occur within Europe, including in Italy, although Spain is still dominated by Lineage 1 (Bakonyi & Haussig, 2020). There is evidence that autochthonous transmission of Lineage 2 has taken place in multiple locations in Europe (Ziegler et al., 2019).

WNV is maintained by a cycle of transmission between bird hosts, primarily of the order Passeriformes, and ornithophilic (bird‐biting) mosquito vectors (Komar et al., 2003). The primary mosquito species contributing to the amplification of WNV in Europe is *Culex pipiens*, although *Culex modestus* and *Culex torrentium* are other vectors present in Europe that can transmit WNV (Balenghien et al., 2006; Leggewie et al., 2016; Turell et al., 2005). *Culex pipiens* is found in Europe as two behaviourally distinct but morphologically identical biotypes; *Culex pipiens pipiens* which is strongly ornithophilic and *Culex pipiens molestus* which is mammalophilic. Hybrids between the two are hypothesized to act as a bridge vector between avian and mammalian populations (Kilpatrick et al., 2005; Vogels et al., 2016).

Mammals, particularly humans and horses, are incidental dead‐end hosts for WNV as they do not produce a sufficient viraemia for onward transmission (Dauphin et al., 2004), with equid cases notifiable to the World Organisation for Animal Health (OIE) (OIE, 2020). Due to vector activity, WNV has a strong seasonality with equid cases in Europe typically reported from mid‐June to mid‐November (Haussig et al., 2018). Infection in humans is asymptomatic in most of the cases, however, a flu‐like syndrome characterized by acute onset fever, fatigue and muscle pain appears in about 20% of the cases (Hayes et al., 2005). Neurological disease in the form of meningitis, encephalitis or paralysis develops in <1% of WNV infections and can lead to death. In horses, neurological disease from WNV has a case fatality rate as high as 30% (Hayes et al., 2005; Paré & Moore, 2018).

European cases of West Nile fever (WNF) have increased in recent years, peaking at 1503 human cases reported in 2018 but remaining high at 463 and 336 in 2019 and 2020 respectively, higher than any years since reporting began in 2010 (European Centre for Disease Prevention and Control, 2020b). WNV is already established in some parts of Europe; phylogenetic analysis indicates that local re‐occurrence, likely due to viral survival in overwintering mosquitoes (Rudolf et al., 2017), is an important factor in European transmission, although the overwintering mechanism is debated and could also be due to other factors, such as virus circulation in non‐migratory birds (Montecino‐Latorre & Barker, 2018). In addition, the annual migration of birds both within and to Europe leads to potential virus re‐introduction and spread in different areas of Europe (Charrel et al., 2003; Monaco et al., 2011; Ziegler et al., 2020). However, it is not always clear which areas in Europe are likely to experience establishment of WNV, which will not have any cases, and which may have repeated re‐introductions but no autochthonous transmission. A map outlining where establishment is possible, given introduction, could help to delineate these areas.

Temperature increases and weather extremes caused by global warming may lead to a change and possibly an increase of areas in Europe with environmental conditions permissive for vector‐borne disease transmission. In addition to WNV, the number of outbreaks of many vector‐borne diseases such as dengue fever, heartworm (*Dirofilaria* spp.) and chikungunya fever have risen in Europe (Genchi et al., 2009; Paz et al., 2013; Rezza et al., 2007; Schaffner & Mathis, 2014). Higher rainfall and warmer temperatures early on in the season create suitable habitats for mosquito breeding, while droughts in summer increase the interaction and likelihood of WNV transmission between mosquitoes and birds (Tran et al., 2014) and these factors are hypothesized to be the reason for the increase in cases in 2018 (Camp & Nowotny, 2020). Global warming also has effects on avian populations with migratory birds from sub‐Saharan Africa now seen in Europe both earlier in the year and further north (Sokolov, 2006). Given the current trends, the threat of WNV in Europe appears to be increasing and early identification of potential risk hotspots for further WNV establishment would support risk‐based surveillance for WNV and prioritization of resources in Europe.

Several methods have been employed to assess the risk of WNV throughout Europe. From the qualitative tool proposed by the European Centre for Disease Prevention and Control (Herrador, 2013) to multivariate models estimating the probability of WNV circulation as a function of different environmental factors affecting mosquito populations (Sánchez‐Gómez et al., 2017; Tran et al., 2014) or Susceptible‐Exposed‐Infective‐Recovered (SEIR) transmission models used to predict WNV spread. Wonham et al. (2004), for example, proposed a simple model with stable mosquito population size to conclude that control of mosquito populations would be a more effective method of disease prevention than bird population control. Bhowmick et al. (2020) incorporated into an SEIR model both temperature‐dependant determinants of mosquito population size and the risk posed by migratory bird populations to provide a risk map of WNV across Germany.

Temperature is a key environmental parameter affecting a range of mosquito traits and therefore significantly influencing transmissibility and risk of WNV establishment. In fact, a number of studies have investigated this relationship by including thermal responses of the individual mosquito traits within the equation for the reproduction number ($R_{0}$) (Kushmaro et al., 2015; Paull et al., 2017; Vogels et al., 2017). These temperature‐dependent versions of $R_{0}$ have been used as a relative measure of establishment risk (Martens et al., 1997; Mordecai et al., 2013; Shocket et al., 2020). $R_{0}$ (or metrics derived from $R_{0}$), are useful measures for establishment risk because $R_{0}$ determines mechanistically if a full vector‐host transmission cycle can take place, a requirement for establishment of a vector‐borne disease.

Indeed, previous studies have used a temperature‐dependant relative $R_{0}$ equation for other vector‐borne diseases to estimate, spatially, the relative risk of establishment after disease introduction (Hartemink et al., 2009; Hartemink et al., 2011; Taylor et al., 2019). Combining this with forecast climate change data has allowed, for example, the prediction of changing malaria distribution patterns across Africa (Moukam Kakmeni et al., 2018). Racloz et al. (2008) combined the temperature‐dependant $R_{0}$ map with existing risk maps of other known geographical and climate risk factors to build a more holistic picture of at‐risk areas for the bluetongue virus in Switzerland. As yet, no map to identify the areas with suitable temperatures for establishment of WNV in Europe, given introduction of the virus, has been created using this technique.

In this study, we build a model to calculate the temperature suitability index for WNV establishment suitability in *Culex pipiens* populations and, using mean monthly temperatures, identify the geographical areas that are suitable for maintenance of infection and/or are at higher risk of WNV establishment following introduction across Europe. Risk maps such as this can be combined with detailed bird distribution and density maps, and other environmental data to identify spatiotemporal areas that are likely to benefit from targeted WNV surveillance and control measures. Furthermore, the model can be combined with predicted temperature increases under different climate change scenarios to determine how areas at risk of WNV establishment could change in the future.

## 2 MATERIALS AND METHODS

$R_{0}$ is most commonly used as an absolute measure of secondary cases generated by an infected individual in a susceptible population (Diekmann et al., 1990). We derive from $R_{0}$ a suitability metric, namely S(T), which determines how suitable each location is for maintenance of circulation and/or establishment of WNV if introduction has occurred. We do this primarily by focusing on the temperature‐dependent factors within $R_{0}$, and scaling it to lie between 0 and 1. While doing so, we remove the bird density within $R_{0}$. This is partly due to the difficulties in assessing densities of bird species across Europe and the need for further research on their respective competence in transmitting WNV (Camp & Nowotny, 2020). As well as removing the differences in birds across Europe, it also removes the differences of introduction or re‐introduction of WNV to different regions through migratory birds. Once this is removed, a likelihood of establishment in mosquito populations, if introduction to an area has occurred, is remaining in our equation for $R_{0}$. This latter term is our suitability metric, a relative measure of establishment risk scaled between 0 and 1. We use this suitability metric S(T), to assess across Europe which areas are relatively more suitable for WNV circulation in *Culex pipiens* (a higher value of S(T)) based on mean temperatures for different months in each area.

We now outline how we derive S(T) from $R_{0}$.The initial $R_{0}$ equation for WNV includes a number of mosquito traits, the majority of which are sensitive to temperature. The temperature‐dependent $R_{0}\;$in our model was derived from a next‐generation matrix (see Supplementary Information) and is similar to that calculated by Dietz (1993) for malaria:

(1) $$R_{0}=\;{\left(\frac{a_{T}^{2}\varphi b_{T}c_{T}e^{-\mu_{T}EIP_{T}}M}{r_{b}B\mu_{T}}\right)}^{1/2},$$

where $a_{T}$ is the mosquito biting rate, proportional to the reciprocal of the gonotrophic (egg‐laying) cycle; φ is the host preference, or probability that the mosquito blood meal is from a bird; $c_{T}\;$is the probability of transmission from bird to mosquito; $b_{T}\;$is the probability of transmission from mosquito to host; $\mu_{T}\;$is the mosquito mortality rate, also used as the mosquito ‘recovery’ rate and $EIP_{T}\;$is the extrinsic incubation period, which is the number of days after feeding on an infectious bird that a mosquito becomes infectious. The component $e^{-\mu_{T}EIP_{T}}$, derived from the Ross‐Macdonald equation, gives the probability that a mosquito will survive the extrinsic incubation period (Smith et al., 2012) while M and B represent the mosquito and bird density respectively. Finally, $r_{b}$ is the infectious period of the bird, included as the inverse of recovery rate. The parameter φ is not necessarily seen in all equations for $R_{0}$ for vector‐borne diseases. Here, and in other equations for WNV (Vogels et al., 2017), it is included since mosquitoes will bite multiple species (birds, humans, horses, other mammals) but for transmission to a mosquito to take place, they first need to bite a bird.

As stated above, the basic $R_{0}$ equation is adapted and extended in line with previous studies (Mordecai et al., 2013; Parham & Michael, 2010; Shocket et al., 2020) by removing the bird density, B, from the equation. Further, due to a lack of comprehensive data regarding mosquito densities across Europe, the mosquito density M is replaced with $M_{T}$ (Equation 2) to include temperature dependant traits affecting the relative mosquito abundance:

(2) $$M_{T}=\frac{V_{T}s_{T}D_{T}F_{T}a_{T}}{\mu_{T}^{2}}\;.$$

Here $V_{T}\;$is the egg viability (the probability of an egg surviving to larval stages); $s_{T}\;$is the probability of a larvae surviving to adult emergence; $D_{T}\;$is the mosquito development rate (the reciprocal of the time between oviposition and adult emergence) and fecundity, as eggs per female per day. In the data, fecundity is presented as eggs/female/gonotrophic cycle ($F_{T}$); conversion to a daily rate requires division by the length of the gonotrophic cycle, equivalent to multiplying by the biting rate ($a_{T}$) as shown in Equation 2. Multiplication by adult lifespan (1/$\mu_{T}$) gives the lifetime egg production. These factors combine to give the number of new adult mosquitoes per day and further multiplication by adult lifespan (1/$\mu_{T}$) gives a measure of the number of mosquitoes at any given time (Mordecai et al., 2013; Parham & Michael, 2010).

With the removal of bird density from the calculation and the estimation of relative temperature‐dependant mosquito abundance $\left(M_{T}\right)$, the new index is no longer an absolute measure of secondary cases but represents a relative measure of establishment risk. We define this to be our suitability metric S(T) which has the following equation:

(3) $$S\left(T\right)=\;C{\left(\frac{a_{T}^{3}\varphi b_{T}c_{T}e^{-\mu_{T}\mathrm{EI}P_{T}}F_{T}V_{T}s_{T}D_{T}}{r_{b}\mu_{T}^{3}}\right)}^{\frac{1}{2}}.$$

The constant C is included in order to normalize this new measure to vary between 0 and 1. This allows comparison of the variation in S(T) more easily across Europe.

As S(T) is a relative metric scaled between 0 and 1, there is no specific value that indicates a threshold for suitability (unlike $R_{0}$). Following on from Ryan et al. (2015) and Taylor et al. (2019), we choose thresholds to denote both higher suitability and a permissive suitability. We choose the median as the threshold for higher suitability as therefore cells with higher than median value (S(T) > 0.5) are considered to have higher suitability. A permissive suitability threshold (S(T)> 0) is used within the Supplementary Information.

### 2.1 Effect of temperature on traits

It is widely acknowledged that mosquito life‐history traits, such as the length of the gonotrophic cycle or development times, exhibit a unimodal response to temperature (Tesla et al., 2018). Similar to other studies that have assessed the temperature dependency of various vector‐borne diseases (Mordecai et al., 2013; Shocket et al., 2018; Shocket et al., 2020; Taylor et al., 2016), for each of the traits included in Equation (3), we quantify this mean relationship with temperature by fitting unimodal responses to laboratory data using the method of non‐linear least squares. We sourced data for each trait across as wide a range of temperatures as possible (Supplementary Information).

We fit three response curves to each of the trait data: a Briére (Briere et al., 1999), a quadratic and a linear response. Unlike a quadratic response, the Briére models an asymmetric response, with the trait increasing steadily at low temperatures but declining sharply as temperature increases past the peak, a known characteristic of arthropod traits (Amarasekare & Savage, 2012; Briere et al., 1999). We also include a linear response, which, although less biologically plausible, may provide a better fit when data are scarce:

(4) $$\mathrm{Linear}:f\left(T\right)=-qT+c,$$

(5) $$\mathrm{Quadratic}:\;f\;\left(T\right)=-q\left(T-T_{\min}\right)\left(T-T_{\max}\right),$$

(6) $${\mathrm{Bri}}^{\prime }\mathrm{re}:\;f\left(T\right)=qT\left(T-T_{\min}\right)\sqrt{\left(T_{\max}-T\right)}.$$

Each of the three responses was fit against the data for each of the traits in S(T) using the *nls* function in R (R Core Team, 2020). The fit was assessed both visually and through use of the corrected AIC (Akaike's Information Criterion), corrected for small sample sizes (Sugiura, 1978). The function for each trait producing the smallest corrected AIC was selected to represent the trait in the S(T) equation. Fitted curves were used to estimate the mean trait value across a temperature range of 0–40°C at increments of 0.1°C. Biological limits were imposed by capping the curves at 0 to prevent negative values and at 1 when the trait is a probability. The values for each trait were then used to estimate S(T) (Equation 3) and produce a plot of S(T) against temperature.

A sensitivity analysis was performed to understand changes in S(T) according to temperature and how this variation is driven by each trait. The change in S(T) (dS/dT) with temperature can be calculated alongside how each of the traits contribute to this change in S(T). This incorporates both the role of the trait in the S(T) equation and the impact of the trait fit against temperature (Supplementary Information).

### 2.2 Data

Results from laboratory and field‐based studies investigating the effect of temperature on the *Culex pipiens* traits in Equation (3) were collected during the literature review and used within the regression fitting for each trait. Details of each data source are shown in Table S1. Unless stated otherwise, all data pertained to *Culex pipiens* and where available, female only data were used as males do not blood feed (Colpitts et al., 2012).

### 2.3 Map construction

Daily temperature data (E‐OBS gridded dataset, v23.1e; Cornes et al., 2018) from 2013–2020 on a spatial scale of 1 km^2^ were used to construct raster maps of monthly mean temperatures across Europe in R. Any day with missing data for each location was excluded from the calculation. There are known issues with the E‐OBS data for areas on the edge of the domain, due to a lack of weather stations. All areas that had data for at least 1 day in each month were included in our maps. The *Culex pipiens* mosquito is widely distributed throughout Europe, although little is known about their distribution on a fine spatial scale; only in Iceland, Svalbard and Jan Mayen have they been declared absent (European Centre for Disease Prevention and Control, 2020a). Therefore, it was assumed that mosquitoes were present in all areas in Europe other than Iceland, Svalbard and Jan Mayen; these areas were excluded from calculations.

The suitability score, S(T), for each raster cell was calculated based on the mean monthly temperature in each cell and Equation (3). All calculations and maps were produced in R (R Core Team, 2020), to visually illustrate the geographical distribution of the most suitable areas (see Supplementary Information). We aggregate these maps into two suitability maps. The first shows the number of months in the year 2020 in which each cell has higher suitability (i.e. S(T)≥0.5). Data from the year 2020 were used due to being the most recent complete year of temperature data, in order to provide a current picture of WNV in Europe. Second, we produce a map showing the average suitability throughout the years 2013–2019. To do this, we calculate for each raster cell the number of months that the cell has suitable values of S(T)≥0.5, for each year from 2013–2019, and then average these values across the 7 years. Alternative versions using the permissive suitability threshold (S(T)>0) are in the Supplementary Information.

### 2.4 Validation with equine case data

Reported equine WNV case data for 2013–2019 were obtained from ADIS (2020) and used to assess the validity of the model. The location of each case (*N *= 12,771) was plotted on the aggregated suitability map and the number of months when that cell had a calculated S(T) ≥ 0.5 was extracted, similar to the method in Taylor et al. (2019) and Ryan et al. (2015). The number of suitable months was plotted against the number of cases and used to determine whether the higher equine disease burden occurred within the areas predicted by the model as having a higher suitability for WNV establishment. Furthermore, we considered the distribution of cells with different months of suitability across Europe in order to assess whether the distribution seen in the validation reflects a true signal of cases occurring in cells with higher suitability, or if it is equivalent to a random distribution of cases across cells in Europe (Supplementary Information).

## 3 RESULTS

### 3.1 Effect of temperature on traits

**FIGURE 1:**
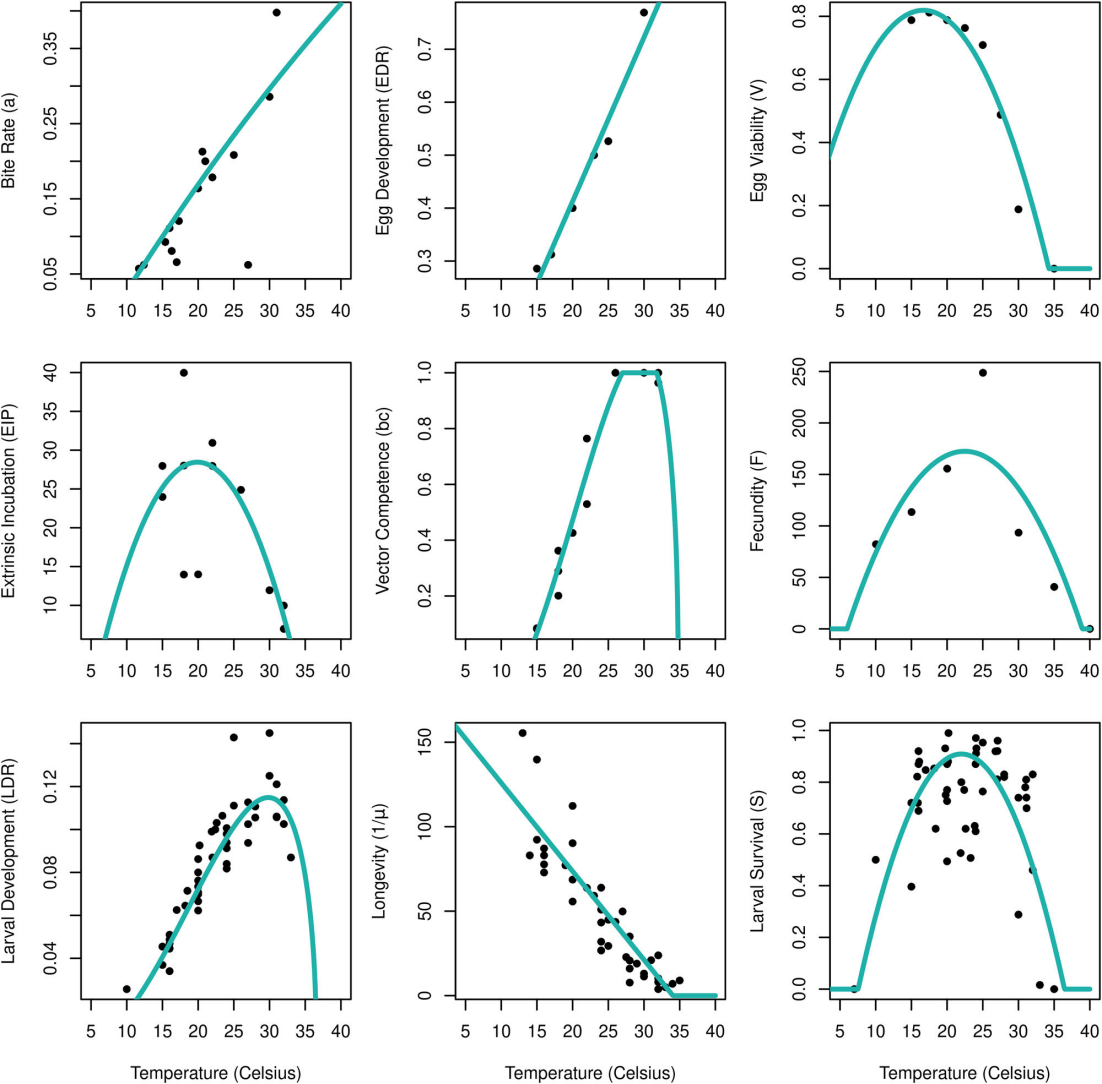
Trait responses to temperature, showing the datapoints and best fitting function curves selected for use in the S(T) model. The mosquito development rate (*D*) in Equation (3) is a combination of the egg development rate (EDR) and the larval development rate (LDR) shown in the figure (see Supplementary Information)

All traits but host preference (φ = 0.81) and avian recovery rate ( $r_{b}\;$= 0.19) were temperature dependant. The datapoints and best fitting function used to represent each are shown in Figure 1 and the parameters for each function are detailed in Table S2. The linear model was the best fit for adult mosquito longevity, reaching 0 days at approximately 34°C. Adult longevity may have remained constant or begun to decrease at lower temperatures than covered by our model; however, *Culex pipiens* enter a state of diapause in winter and are known to survive for a number of months at low temperatures, so no additional limitations were enforced (Liu et al., 2016; Mitchell & Briegel, 1989). A linear fit was also best for the egg development rate (EDR).

Extrinsic incubation period ($EIP_{T}$), egg viability ($V_{T}$), fecundity ($F_{T}$) and larval to adult survival probability ($S_{T}$) were all best fitted with quadratic curves; the permissive minimum temperatures ranged from −1°C to 7.54°C and the maximum from 34°C to 39°C. The optimal temperatures were between 15°C and 25°C. A quadratic curve was also the best fit for the biting rate ($a_{T}$), although the curve is still increasing past 40°C. The collated data only showed an increase with temperature up to 30°C, due to a lack of data on biting rate at higher temperatures found in the literature. The biting rate would be expected to decline past 30°C, but the data are lacking. However, other traits, such as mortality rate ($\mu_{T}$), in the $R_{0}$ equation will enforce this decline instead.

Vector competence ($bc_{T}$) and larval development rate (LDR) were best fitted by asymmetrical Briére functions with optimal temperatures between 28°C and 31°C. Longevity ($1/\mu_{T}$), larval to adult survival ($S_{T}$) and larval development rate ($D_{T}$) had the most datapoints associated with them and therefore less uncertainty around the choice of function, although this was not quantified as part of the study.

### 3.2 S(T) suitability metric

The temperature‐dependent S(T) as in Equation (3), scaled from 0 to 1 (with 1 being the maximum suitability) is a bell‐shaped curve (Figure S1) which has optimal temperature for WNV establishment of 23.7°C. The lower and upper thermal limits were 14.0°C and 34.3°C. The lower thermal limit is imposed by vector competence which remains at 0 below 14.0°C and the upper limit by extrinsic incubation period which reaches 0 at 34.3°C.

### 3.3 Sensitivity analysis

**FIGURE 2:**
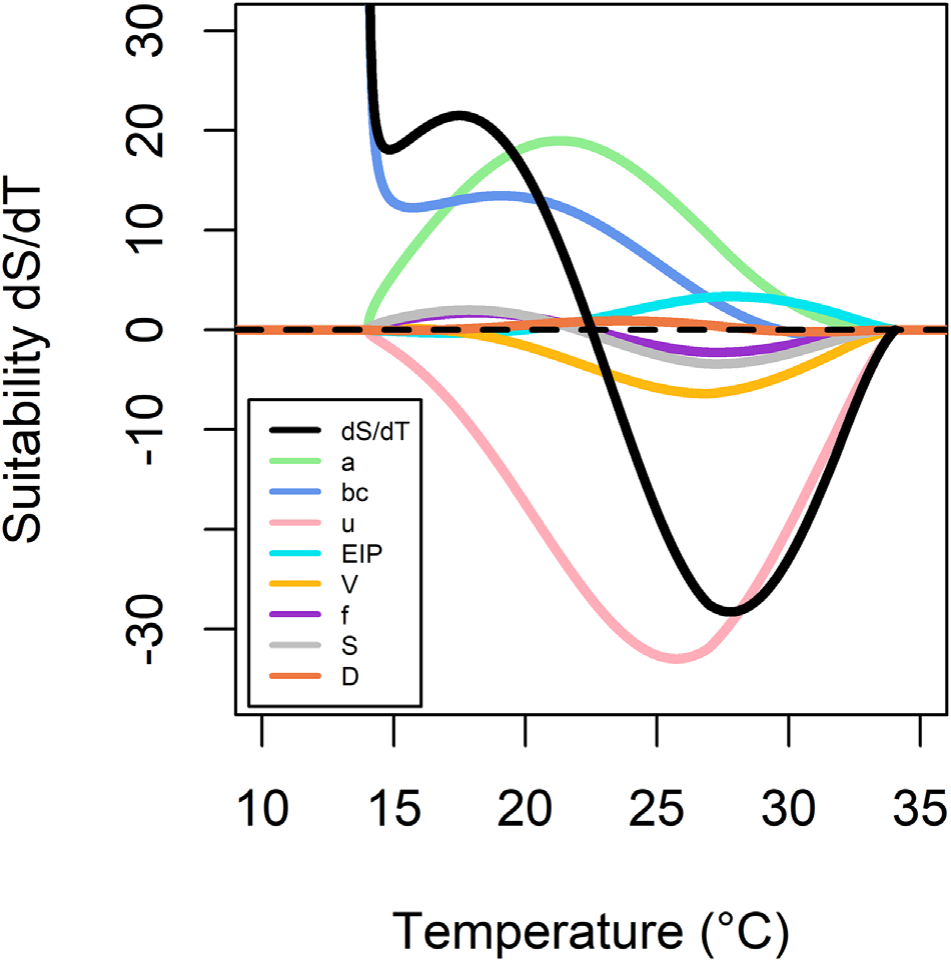
The sensitivity of S(T) to changes in each of the temperature‐dependant traits by considering the derivative, dS/dT, of S(T). The further dS/dT is from the *x*‐axis, the steeper the increase or decrease in S(T). Each coloured curve represents the impact that parameter has on the change in S(T), calculated for each parameter as dx=dS/dxdx/dT. The temperatures at which the curves appear to tend towards infinity are when S(T) approaches 0

Figure 2 shows how S(T) changes with temperature; the further dS/dT is from the *x*‐axis indicates how quickly S(T) is growing or declining with temperature, depending on whether dS/dT is positive or negative respectively. Figure 2 shows that initially S(T) is increasing very fast but this rate of increase slows down until it crosses the *x*‐axis – the temperature at which dS/dT crosses the *x*‐axis is the temperature of peak S(T). Furthermore, the coloured lines for each parameter indicate how much the change in dS/dT is attributable to that parameter. Thus, the rising gradient of S(T) seen with increasing temperatures up to 20°C is predominantly caused by vector competence (bc) and biting rate (a) as they have the highest positive values. Above 20°C, S(T) continues to increase but at a slower rate, despite the biting rate and vector competence continuing to increase with temperature. This slowed increase and subsequent decrease in S(T) is primarily driven by adult mortality (μ), which overall has the largest impact onS(T) (furthest from the *x*‐axis). This parameter has a negative impact on the suitability because as mortality of mosquitoes increases, they are less likely to complete the extrinsic incubation period and transmit WNV, and therefore the suitability of an area with higher mortality will be lower. Other traits also saw negative changes with increasing temperature above 19°C; these were egg viability (V) and, beyond 21°C, fecundity (F) and larval survival probability (s). Overall, this figure indicates that vector competence (bc), bite rate (a) and extrinsic incubation period (EIP) are the parameters driving a higher suitability, as they have the highest positive values across all parameters.

### 3.4 Geographical distribution

**FIGURE 3:**
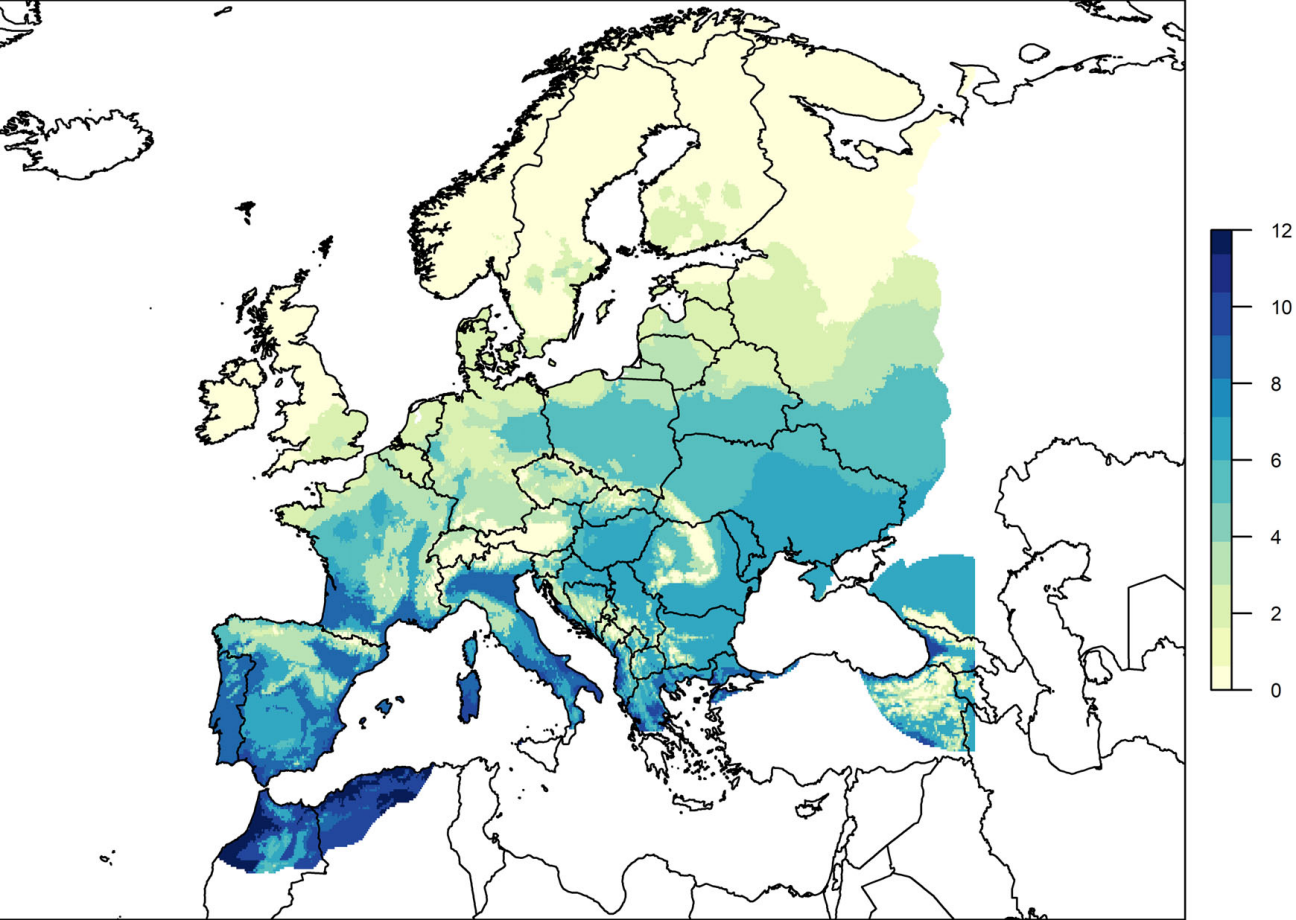
The number of months in 2020 that demonstrated a higher suitability for WNV establishment (S(T)≥ 0.5). The European coastline and country border shapefiles were from EEA (2015) and Natural Earth (https://naturalearthdata.com), respectively

The aggregate suitability map for 2020 is plotted across Europe in Figure 3. This indicates the number of months a location spends with a temperature dependant S(T)≥0.5 out of the 12 months of 2020. The values of S(T) for each raster cell across Europe for each month of 2020, and an aggregate suitability map for a permissive S(T)> 0 can be found in the Supplementary Information.

The majority of southern Europe including areas of Spain, Portugal, southern France, Italy, Greece, Romania, Hungary, Bulgaria and Turkey shows prolonged higher suitability for WNV. Portugal, Greece, Turkey, southern Spain and Italy in particular showed upwards of 8 months of higher establishment suitability. Only very northern Europe and areas at high altitudes did not display any average monthly temperatures with higher thermal suitability for WNV establishment. June through to August saw the most widespread establishment suitability (Figure S2). Almost all of Europe displayed WNV permissive monthly temperatures for at least 1 month in 2020 (Figure S3). However, some locations in central Spain, Italy, Greece and southern France experienced monthly average temperatures higher than the optimal establishment temperature (temperature at peak S(T)) in July and August (although the areas still had values of S(T)≥0.5), indicating that if environmental temperatures increase with global warming, these areas may become too hot for transmission.

**FIGURE 4:**
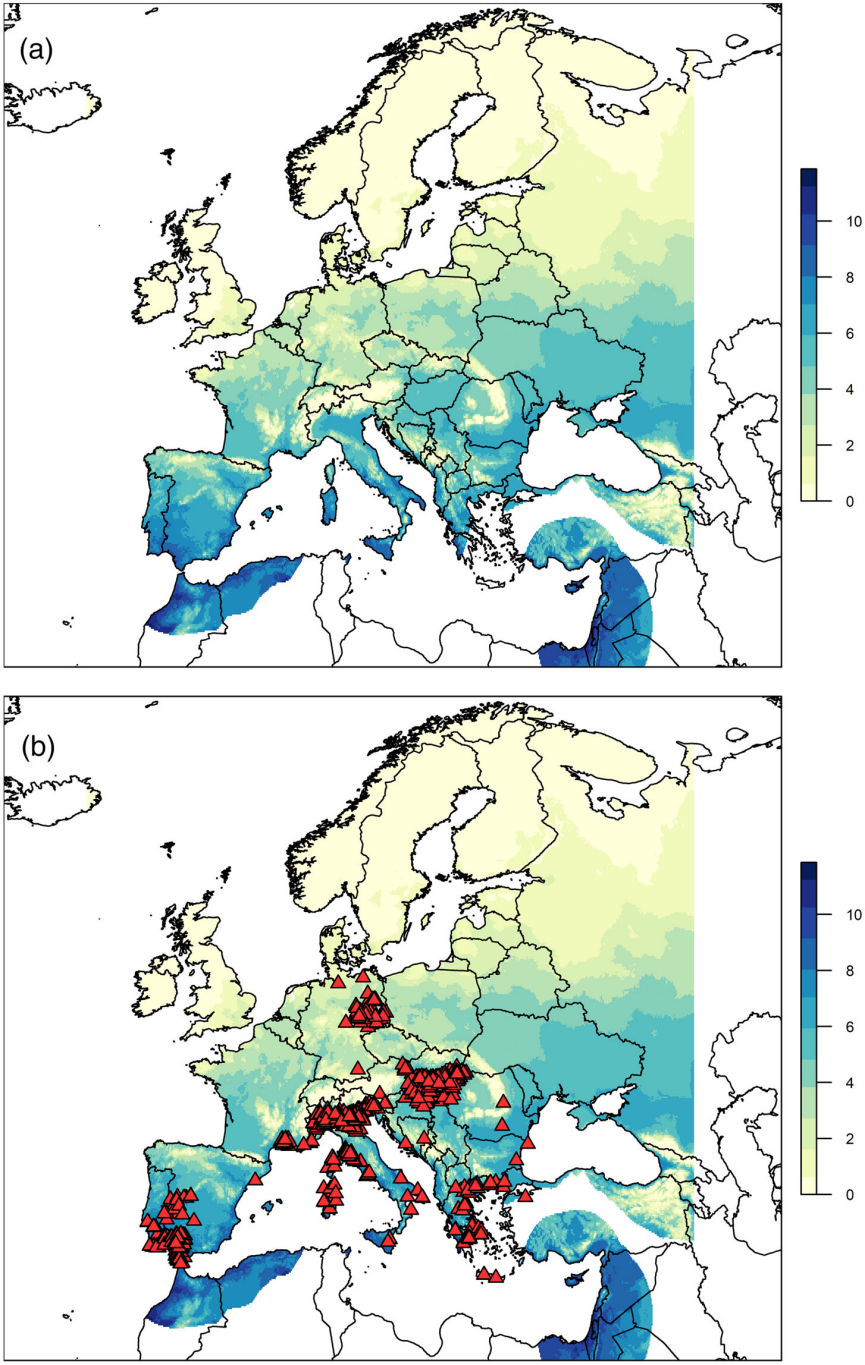
The number of months that demonstrated a higher suitability (S(T)≥0.5), calculated for each year from 2013 to 2019 and then averaged, is plotted in a and b. In b, the locations of cases of equine WNV extracted from ADIS (2020) from 2013 to 2019 are also plotted as red triangles

When considered as an average of suitability from 2013 to 2019 (Figure 4), the map shows a similar picture to the 2020 year on its own. However, in the 2020 map there are more areas with higher suitability, suggesting that 2020 was warmer and more suitable for WNV transmission than the average over 2013–2019. However, this does not mean that 2013–2019 were not highly suitable. When considered as individual years (Figure S4), the years 2017 and 2019 seem to have the most suitability within the range of 2013–2019.

### 3.5 Validation with equine case data

**FIGURE 5:**
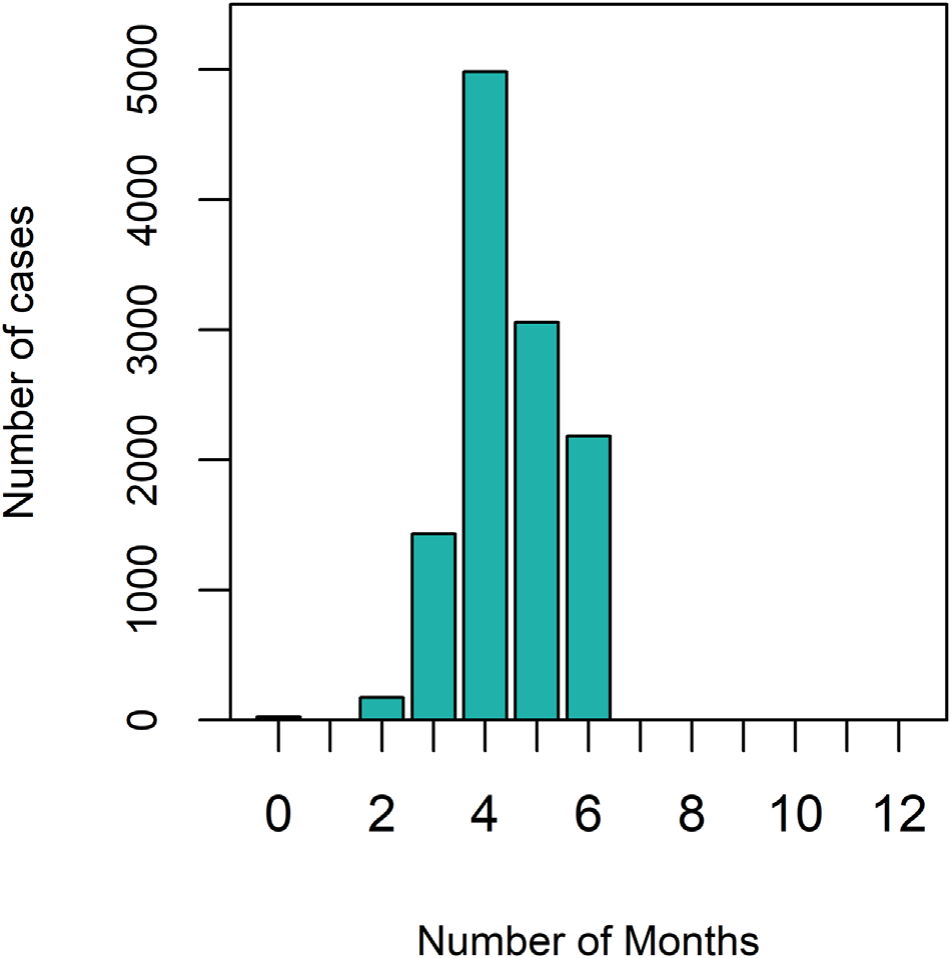
The number of equine cases from 2013 to 2019 that occurred in areas with 0–12 months of higher suitability (S(T)≥0.5). Equine cases (*N* = 12,771) were extracted from ADIS (ADIS, 2020)

The locations of equine cases that occurred in 2013–2019 (*N* = 12,771) are plotted on top of the suitability map in Figure 4b, indicating that cases do predominantly occur in areas that have higher suitability for many months of the year. In fact, the majority (98%) of the equine cases from 2013 to 2019 occurred in locations that had average temperatures with higher suitability (S(T) ≥ 0.5) for at least 3 months of the year (Figure 5). Forty‐two per cent of cases occurred in locations that had higher suitability for 4 months of 2020, but no cases occurred in locations with more than 6 months of higher suitability. This is primarily driven by the fact that only 1.6% of raster cells within our map that have this higher suitability, but also because the areas with higher suitability all year round are located in northern Africa, but our equine cases are restricted to Europe. The cases that occurred with 0 averaged months of higher suitability were in one single location in Romania, which had S(T)≥0.5 for 1 month in 1 year out of 2013–2019 (the year when the cases occurred) and at least 3 months of permissive suitability on average. When considering the permissive suitability, all cases occurred in areas with between 3 and 10 months of suitability, averaged across 2013–2019, with 35% of cases occurring in locations with 6 months of permissive suitability (Figure S5).

## 4 DISCUSSION

In this study, we derive a suitability metric, S(T) from the basic reproduction number, $R_{0}$, assess how this metric varies with temperature and then use S(T) to explore the effect of environmental temperature on the risk of WNV establishment. Thermal response data for a number of *Culex pipiens* traits were combined mechanistically to produce a suitability of establishment model that, when plotted with European temperature data for 2020, allowed identification of a prolonged and widespread suitability for WNV across Europe. Areas of higher suitability for numerous months – that also see high avian traffic from Africa – were southern Spain, Italy, Greece and Turkey.

Our study complements and extends the existing literature by bridging the gap between theoretical WNV $R_{0}$ models and spatial risk assessments. The model estimated the optimal S(T) of WNV in *Culex pipiens* mosquitoes to be at 23.7°C which is within 1.2°C of the 24.5°C optimum estimated by Shocket et al. (2020) and the 24.9°C optima of Paull et al. (2017). It is however lower than predicted by other studies; Vogels et al. (2017) predicted an optimal temperature of 28°C and Kushmaro et al. (2015) suggests that $R_{0}$ will continue increasing up to 35°C in *Culex pipiens* populations. All of these studies other than Paull et al. (2017) were developed using similar techniques and data sources. Our low optimal temperature leads to the counterintuitive result that some areas of southern Europe have temperatures higher than the optimal, and in fact global warming may lead to these areas becoming less suitable for WNV transmission. Our sensitivity analysis indicated that adult mortality rate is the main driver for S(T) reduction at higher temperatures; therefore, this may be an area to focus further research on to understand establishment of WNV at these higher temperatures. When our risk maps were compared to those created through different methodologies, some similarities were identified; Sánchez‐Gómez et al. (2017) created a risk map of WNV in Spain based on 2015 avian, equid and human case data and the areas identified as highest risk are almost identical to those with 6 months or more of high suitability in our model. The risk map created by Tran et al. (2014), which used 2013 surface water and temperature anomalies, identified hotspots further east in Europe, in Greece, Hungary and northeast Italy. Similarly, the areas of Greece and Italy were also identified as prolonged high risk in our model, indicating the same areas to be at high risk year on year. Further, our risk maps created separately from 2013 to 2020 highlight that the same areas are at risk each year.

However, as well as predicting similar areas of high suitability to previous models, our model extends the regions that are potentially at risk of establishment should introduction occur. This is because we used a mechanistic approach rather than relying on species distribution models based on where cases have already occurred. This leads to better prediction of establishment suitability in areas which have not already had cases. For example, our model predicts numerous months of potential transmission in areas further north in Europe, such as the Netherlands, the United Kingdom, Germany and Poland, which is not the case for Tran et al. (2014). WNV cases have occurred in northern Germany each year since 2018 and the Netherlands as of October 2020 (Vlaskamp et al., 2020), indicating that establishment is possible. In fact, when considering the yearly maps (Figure S4), a pattern emerges of Germany becoming more suitable from 2013 with most of Germany having 1 or 2 months of suitability above 0.5, to 2019 with the majority of Germany having 4 months of higher suitability. The larger area of establishment suitability arises in our model due to the lower temperature for optimal S(T), suggesting that WNV can exist in an autochthonous cycle in these more northerly regions of Europe.

To assess whether the model could predict the locations of WNV outbreaks, we compared the locations of equine WNF cases from 2013 to 2019 with the predicted temperature suitability maps (Figure 5). Aggregate data were used as there is no consensus on the time lag between establishment suitability and WNF case confirmation and use of overall data avoids unnecessary assumptions (Paull et al., 2017; Shocket et al., 2018). Validation of the model with equine case data showed that 98% of cases occurred in locations that spent at least 3 months with higher WNV establishment suitability, enough time for the WNV transmission cycle to occur multiple times. This is consistent with the large outbreak in Germany, suggesting that the majority of Europe with similar risk patterns could be susceptible to outbreaks. Locations that our model predicts to have spent at least 6 months in permissive temperatures (S(T)> 0) such as southern Spain, Italy and Greece were home to 70% of the equine WNF cases seen in 2013–2019.

Equine case data of WNV is restricted to those horses which developed neurological disease and then were reported to the OIE. Not all horses infected with WNV will develop disease, nor will all horses with the disease have clear signs that are recognized as WNV (Castillo‐Olivares & Wood, 2004). Therefore, it is likely that the equine case data are missing less severe cases and other unreported cases. However, there is no reason to believe these unreported cases would be more likely to occur in different areas to the reported cases. It is possible for horses to be vaccinated against WNV (Seino et al., 2007), but, according to the OIE WAHIS database (OIE, 2021), the only country in Europe which has reported any official vaccination for WNV since 2006 is the United Kingdom, although reporting is not mandatory. Furthermore, horses are not homogeneously distributed across Europe leading to biases in where cases are reported. However, according to World Horse Welfare and Eurogroup for Animals (2015), the top three countries with the highest number of horses are France, Romania and the United Kingdom, which are not the same countries with most cases, suggesting that the number of cases reported is not primarily correlated with where horses are. This suggests that although there are some issues with the equine case data, it is unlikely to have a significant impact on our validation.

We used equine case data to validate the maps due to the easy availability of these data across Europe at a fine spatial scale (i.e., latitude and longitude of case locations). Human case data were not so appropriate as a validation dataset because this is only provided on a NUTS3 level (European Centre for Disease Prevention and Control, 2020b) and therefore we would have needed to aggregate our suitability maps spatially to the level of the human case data. When compared qualitatively, human cases since 2011 have occurred in southern Spain and Portugal, Germany, northern Italy and across much of the Balkans. This is in line with both the locations for the horse cases and the suitability outputs we produced.

While temperature is known to be an important factor in the epidemiology of WNV, many other environmental factors affect transmission including surface water coverage (i.e. wetlands), land usage and altitude that were not explored in this study (Medlock & Leach, 2015; Paz et al., 2013; Tran et al., 2014). The individual mosquito trait responses to these factors have not been comprehensively explored in experimental studies and their effect on mosquito populations is more easily investigated through other methodologies such as species distribution modelling (Paz, 2015).

Equine cases of WNV are clustered and remain in the same areas year on year, once they occur within a region. This suggests that overwintering/establishment of the cases, through birds or mosquitoes, is a relevant factor in the distribution of WNV cases. However, new introductions into new regions of Europe could also occur via bird migration. This could be via birds migrating into Europe from sub‐Saharan Africa, where WNV is also present, or from migration of birds within Europe. Indeed, phylogenetic analysis suggests cases of WNV are closely linked to clusters in other locations in Europe (Chaintoutis et al., 2019). An improved prediction for WNV could be achieved by including distribution, migration patterns and density of relevant bird species across Europe. Bird migration patterns from outside Europe show a seasonal pattern of avian hosts arriving in the spring from sub‐Saharan Africa, often arriving first to those areas of Europe which are identified in our model as a high suitability of establishment. The influx of birds could both bring in new cases and lead to spread of the virus that is already known to be autochthonous in some of these high traffic regions. These ‘bottle‐neck’ areas of southern Spain, Italy, Greece and Turkey, which also have higher suitability, are therefore at the highest risk of WNV for re‐introduction (Busse et al., 2014). Bird migrations patterns within Europe are less well known and may not take place only in spring. Furthermore, the role of different bird species as amplifying hosts is not well understood, as well as distributions of these different species across Europe, and so further work is needed in this area (Camp & Nowotny, 2020). By not including these factors of bird distribution, our model primarily considers how establishment across Europe is driven by mosquito species. Therefore, our suitability maps are most useful to inform risk‐based active surveillance of mosquitoes.

A more detailed risk of introduction map, based on factors such as local and long‐distance bird migration, prevalence of WNV in birds, distribution, density and WNV competence of various bird species and movement of vectors to new areas has not been produced to our knowledge, especially not on a fine spatial scale. An important next step is to combine a risk of introduction map such as this with our risk of establishment map to produce an overall risk map identifying the most important hotspots across Europe for WNV. This could then be used to target surveillance, vaccination and other prevention and control measures, to specific locations on a fine spatial scale. The risk of establishment on its own allows for focusing prevention and control measures that are specifically tailored to the mosquitoes, the determining aspect of this risk.

Our suitability model made the assumption that no bird‐to‐bird or vertical mosquito transmission took place and that the establishment was occurring in fully susceptible populations. Bird‐to‐bird and vertical mosquito transmission are thought to represent a small proportion of transmission events (Hartemink et al., 2007). Bird immunity to WNV is possible, and bird populations in areas of Europe with cases have been shown to have low levels of seropositivity in local studies (Pallari et al., 2021; Bażanów et al., 2018). Bird immunity would have most impact on our model by reducing the vector competence parameter. As shown by the sensitivity analysis (Figure 2), this parameter primarily affects the suitability metric at lower temperatures. Therefore, bird immunity would be more likely to reduce the suitability for establishment at these lower temperatures. Similarly, Kushmaro et al. (2015) considered endemic and newly emerged regions for WNV and estimated that the basic reproduction number, $R_{0}$, would increase above 1 at lower temperatures for newly emerged regions.

The sensitivity analysis investigated the effect of changes to each trait to the S(T) output and indicated that the trait most influential to S(T) was the mosquito mortality rate followed by the mosquito‐biting rate. The sensitivity analysis therefore suggests that the control strategies to decrease the establishment suitability of WNV which target higher adult mosquito mortality or decreased mosquito biting rate could be most successful, if the control strategies are able to effectively impact those parameters. More data to illustrate the relationship between mortality rate and temperature would have the greatest impact on the accuracy of S(T). The other two traits with a large impact were biting rate and vector competence; the temperature dependency of the biting rate, in particular, would benefit from further research as it is difficult to measure and there were limited data available.

As the spread of vector borne diseases in Europe increases with the changing climate, adaptations to existing disease surveillance and control strategies in Europe need to be made. WNV is a disease of both human and veterinary importance that already has a high suitability for establishment across much of Europe. This mechanistic model estimates areas of potential establishment suitability of WNV if an introduction event were to occur, independent of where cases have occurred previously. The method also allows the understanding of potential mitigation measures to reduce this suitability through the sensitivity analysis, which indicated targeting mosquito mortality or biting rate. Lastly, the model can be used in conjunction with forecast climate data and models exploring other environmental risk factors to locate future risk hotspots around Europe and target surveillance and public health messages accordingly. Given the importance of tackling diseases before they can establish and spread, the use of models, surveillance and other techniques to locate potential disease hotspots and target introduction prevention are of key significance.

## CONFLICT OF INTEREST

The authors declare no conflict of interest.

## ETHICS STATEMENT

No ethical approval was required as this article produced no original research data.

## Supporting information

Supporting Information

## Data Availability

Data sharing is not applicable to this article as no new data were created or analysed in this study.
