# Supplementary material for: Modelling the temperature suitability for the risk of West Nile Virus establishment in European Culex pipiens populations
Source: Transbound Emerg Dis. 2022 Mar 28;69(5):e1787–99. doi: 10.1111/tbed.14513 (PMC9790397; doi:10.1111/tbed.14513)
Supplement: Supplementary file 1 — Supporting Information [file TBED-69-e1787-s001.pdf]

Modelling the temperature suitability for the risk of West Nile virus establishment in Europe in *Culex pipiens* populations

Supplementary Information

Gabriella Di Pol[1], Matteo Crotta[1] and Rachel A. Taylor[2]

[1] Veterinary Epidemiology, Economics and Public Health Group, Department of Pathobiology and Population Sciences, Royal Veterinary College, UK

[2] Department of Epidemiological Sciences, Animal and Plant Health Agency, UK

Corresponding author: Rachel A Taylor
APHA-Weybridge
New Haw, Addlestone, KT15 3NB, Surrey, UK
Rachel.taylor@apha.gov.uk

1. Additional Methodological Details

R0 Equation Derivation:

The equation for R_0 is calculated from a next generation matrix adapted from Vogels et al. (2017), where k_{mh} indicates the number of mosquitoes that acquire WNV from an infected host (bird) and k_{hm} indicates the number of hosts (avian, equid or human) that acquire infection from a mosquito. It is assumed that no host to host or mosquito to mosquito transmission takes place. Subscript T indicates that a trait is temperature dependant.

$$\begin{pmatrix} 0 & k_{mh} \\ k_{hm} & 0 \end{pmatrix} = \begin{pmatrix} 0 & \dfrac{a_T \varphi c_T M}{r_b B} \\ \dfrac{a_T b_T e^{-\mu_T EIP_T}}{\mu_T} & 0 \end{pmatrix} \qquad \text{Equation S.1}$$

Birds are the primary route of WNV introduction as mammalian hosts do not cause onward transmission. Hence the traits included in the k_{mh} equation pertain only to avian hosts and are as follows: the mosquito biting rate, proportional to the reciprocal of the gonotrophic (egg-laying) cycle (a_T); the host preference, or probability that the mosquito bloodmeal is from a bird (φ); mosquito density (M); bird density (B) and the infectious period of the bird, included as the inverse of recovery rate (r_b). These five traits combine to give the number of bites that a WNV positive bird will receive whilst infectious. The probability of transmission from bird to mosquito (c_T) is also included.

k_{hm} includes: the mosquito biting rate (a_T); the probability of transmission from mosquito to host (b_T); the mosquito mortality rate (μ_T), also used as the mosquito 'recovery' rate; and the extrinsic incubation period, which is the number of days after feeding on an infectious bird that a mosquito becomes infectious (EIP_T). The combination $e^{-\mu_T EIP_T}$, derived from the Ross-Macdonald equation, gives the probability that a mosquito will survive the extrinsic incubation period (Smith et al., 2012).

Combination of the next generation matrix into an equation for the value of R_0 is calculated via the method described by Diekmann and Heesterbeek (2000).

Data

Table S1: *The sources compiled to establish the values of each trait used in the final R_0 model. Where study populations were separated by sex, female mosquito data was used. The traits are given per day unless specified. In the event that multiple scenarios were investigated alongside temperature, such as different viral strains, altitudes or captive vs field bred mosquitoes, all were included in the fit to resemble natural variation.*

Trait	Trait Description	Sources used
Biting Rate (a_T)	Reciprocal of the gonotrophic cycle (time between feeding and egg laying) was taken to be proportional to bite rate.	Madder et al. (1983)
Host Preference (φ)	Probability that the mosquito blood meal is a bird. Weighted mean was calculated based on mosquito sample size in each of the studies.	Hamer et al. (2009), Osório et al. (2014), Fritz et al. (2015)
Vector Competence (bc_T)	The probability of transmission from bird to mosquito to host. See below.	Dohm et al. (2002), Kilpatrick et al. (2008)
Mortality Rate (μ_T)	Reciprocal of adult longevity. Where Kaplan-Meier survival plots were given, the median survival time was taken.	Andreadis et al. (2014), Ciota et al. (2014), Ruybal et al. (2016)
Extrinsic Incubation Period (EIP_T)	Number of days from infectious feed to the highest proportion of mosquitoes showing disseminated infection.	Dohm et al. (2002), Kilpatrick et al. (2008)
Recovery Rate of Birds (r_b)	Inverse of the infectious period of the bird. See below.	Komar et al. (2003), Lim et al. (2015)
Egg Viability (V_T)	Probability of an egg surviving to larval stages. This data was from the species *Culex modestus*.	Spanoudis et al. (2018)
Fecundity (F_T)	Eggs per female per gonotrophic cycle. See below.	Shocket et al. (2020)
Larval to adult survival probability (s_T)	Probability of larvae surviving to adult stages.	Madder et al. (1983), Loetti et al. (2011), Ciota et al. (2014), Ruybal et al. (2016)
Mosquito development rate (D_T)	Reciprocal of total development time through egg and larval stages. See below.	Madder et al. (1983), Loetti et al. (2011), Ciota et al. (2014), Ruybal et al. (2016)

Vector Competence (bc_T) was extracted from the data as the highest proportion of mosquitoes with disseminated infection for each temperature. Mosquitoes with disseminated infection are thought to reasonably approximate those transmitting WNV (Turell et al., 2001). This also provided us with the corresponding number of days post infectious feed (EIP).

Recovery Rate of Birds (r_b) was for passerine species only. Birds with viraemia greater than 10^5PFU/mL were considered infectious to *Culex pipiens* (Turell et al., 2000). A mean for the two studies weighted on sample size was calculated.

Fecundity (F_T) data was not readily available; data points were taken from Shocket et al. (2020) originally published in Li et al. (2017). No weightings were available and data was from the subspecies *Culex pipiens pallens*.

Mosquito development rate (D_T) was presented separately for egg and larval stages. Egg development rate was from Madder et al. (1983). Larval development rate (LDR) and egg development rate (EDR) were combined as follows:

$$\mathrm{D} = \frac{LDR.EDR}{\mathrm{LDR} + \mathrm{EDR}}.$$

Sensitivity analysis:

A sensitivity analysis is carried out to assess how the traits and overall $S(T)$ are affected by changes in temperature. This is done by differentiating $S(T)$ with respect to temperature using the following equation:

Equation S.2

$$\frac{\partial S}{\partial T} = \frac{\partial S}{\partial a}\frac{\partial a}{\partial T} + \frac{\partial S}{\partial bc}\frac{\partial bc}{\partial T} + \frac{\partial S}{\partial \mu}\frac{\partial \mu}{\partial T} + \frac{\partial S}{\partial EIP}\frac{\partial EIP}{\partial T} + \frac{\partial S}{\partial F}\frac{\partial F}{\partial T} + \frac{\partial S}{\partial V}\frac{\partial V}{\partial T} + \frac{\partial S}{\partial s}\frac{\partial s}{\partial T} + \frac{\partial S_0}{\partial D}\frac{\partial D}{\partial T}.$$

Here $\frac{\partial S}{\partial x}$ is the partial derivative of $S(T)$ with respect to trait x and $\frac{\partial x}{\partial T}$ is the partial derivative of x with respect to temperature. The contribution of individual traits to the $S(T)$ temperature sensitivity can be assessed using these components of Equation S.2, for example the temperature sensitivity arising through biting rate (a) is shown by the equation $\frac{\partial S}{\partial a}\frac{\partial a}{\partial T}$. This influence of each trait on the sensitivity of $S(T)$ to temperature is then plotted in Figure 3 in the main text alongside the overall sensitivity of$S(T)$.

2. Additional Results

This section contains a table showing the fit for the thermal response curves for each trait (Table S2), the plot of our suitability metric against temperature (Figure S1) as well as additional maps and validation. In particular, the maps for each month in 2020 showing the calculated $S(T)$ (Figure S2); an aggregated map showing the number of months Europe spends in permissive temperatures for WNV establishment ($S(T)$>0) in 2020 (Figure S3); and the aggregated maps for each year of 2013 to 2019 for the number of months with higher suitability ($S(T) \geq 0.5$), with the cases from each year plotted on top (Figure S4). For the validation, we also provide the number of reported equine WNV cases in 2013-2019 located in areas of varying numbers of months with permissive temperatures for WNV (Figure S5); and a distribution of the number of months of suitability for all cells in Europe compared against those cells with cases, for both the higher suitability threshold (Figure S6) and the permissive threshold (Figure S7).

Table S2*: Details of the functions used to plot each trait against temperature. The functions are detailed in Equations 4-6 in the main text. Where constant is listed under function, the trait was incorporated independent of temperature.*

Trait	Function	Function Parameters	
Biting Rate<br>(a_T)	Quadratic	$\boldsymbol{q}$<br>$\boldsymbol{T_{min}}$<br>$\boldsymbol{T_{max}}$	6.74 x10^{-5}<br>8.72<br>250.60
Host Preference<br>(φ)	Constant		0.81
Vector Competence<br>(bc_T)	Briére	$\boldsymbol{q}$<br>$\boldsymbol{T_{min}}$<br>$\boldsymbol{T_{max}}$	1.03 x10^{-3}<br>14.02<br>34.76
Adult longevity<br>$(1/\mu_T)$	Linear	$\boldsymbol{q}$<br>$\boldsymbol{c}$	5.24<br>178.32
Extrinsic Incubation Period<br>(EIP_T)	Quadratic	$\boldsymbol{q}$<br>$\boldsymbol{T_{min}}$<br>$\boldsymbol{T_{max}}$	0.14<br>5.43<br>34.36
Recovery Rate of Birds<br>(r_b)	Constant		0.19
Egg Viability<br>(V_T)	Quadratic	$\boldsymbol{q}$<br>$\boldsymbol{T_{min}}$<br>$\boldsymbol{T_{max}}$	2.65 x10^{-3}<br>-0.91<br>34.26
Fecundity – Eggs per female per gonotrophic cycle<br>(F_T)	Quadratic*	$\boldsymbol{q}$<br>$\boldsymbol{T_{min}}$<br>$\boldsymbol{T_{max}}$	0.64<br>5.99<br>38.94
Larval to adult survival probability<br>(s_T)	Quadratic	$\boldsymbol{q}$<br>$\boldsymbol{T_{min}}$<br>$\boldsymbol{T_{max}}$	4.35 x10^{-3}<br>7.54<br>36.45
Larval to adult development rate<br>(LDR)	Briére	$\boldsymbol{q}$<br>$\boldsymbol{T_{min}}$<br>$\boldsymbol{T_{max}}$	6.08 x10^{-5}<br>5.41<br>36.55
Egg development rate<br>(EDR)	Linear	$\boldsymbol{q}$<br>$\boldsymbol{c}$	0.031<br>-0.21

*The corrected AIC was slightly lower for a linear fit, however upon visual inspection the data shows a clear unimodal trend and a quadratic curve was selected to improve biological representation.

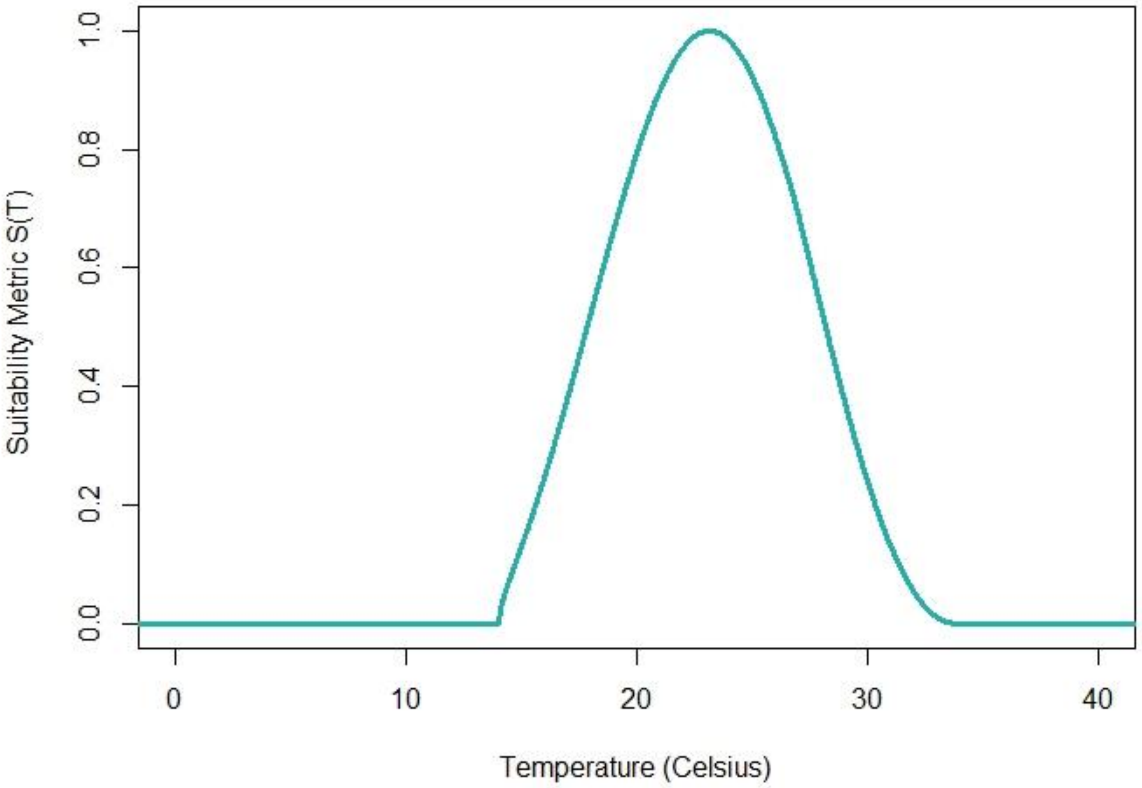

Figure S1: *A plot of the suitability metric* $S(T)$ *against temperature showing a peak at* 23.7°C.

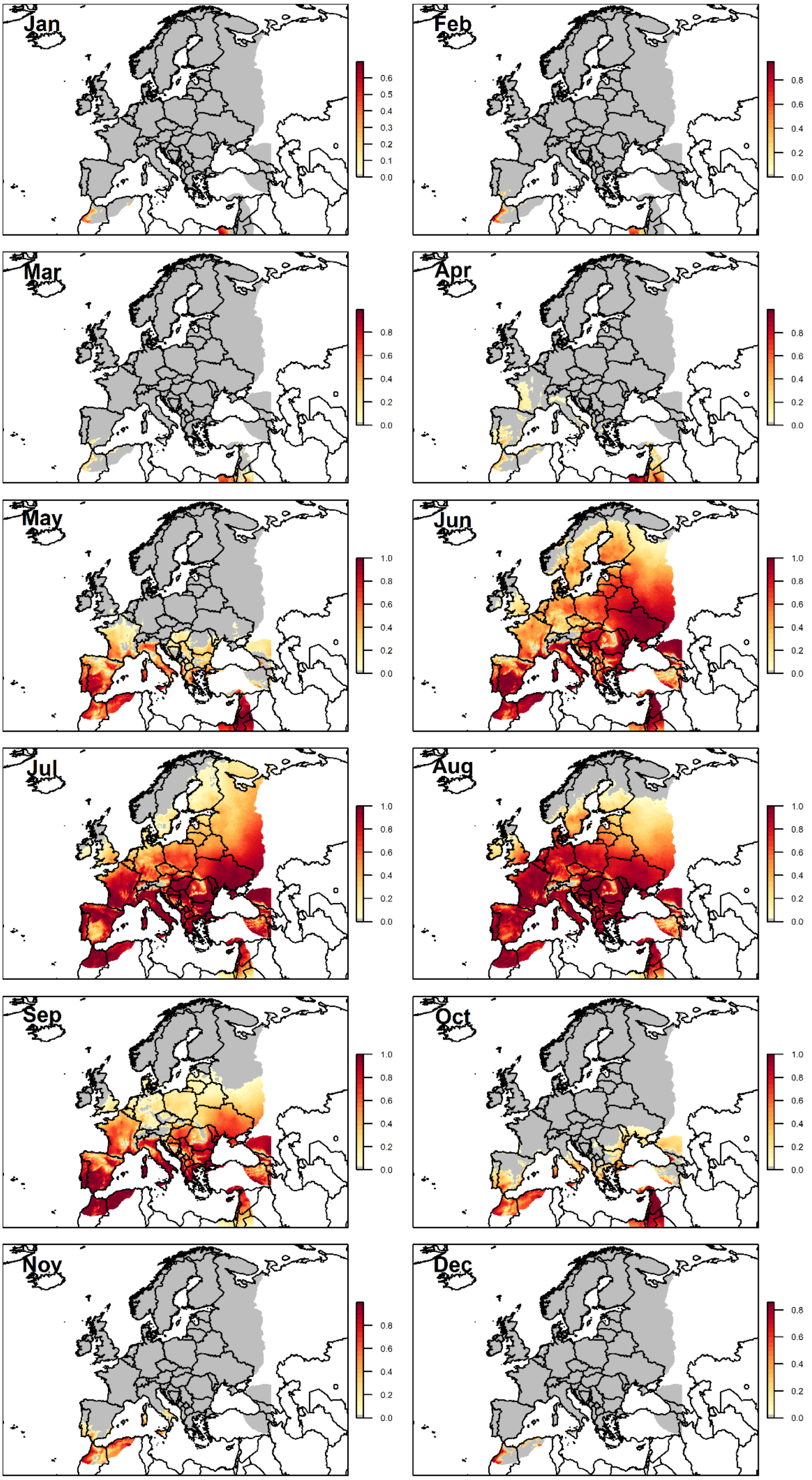

Figure S2: *The temperature dependent value of* $S(T)$ *across Europe for each month of 2020 from January to December at a* $1km^2$ *cell level*

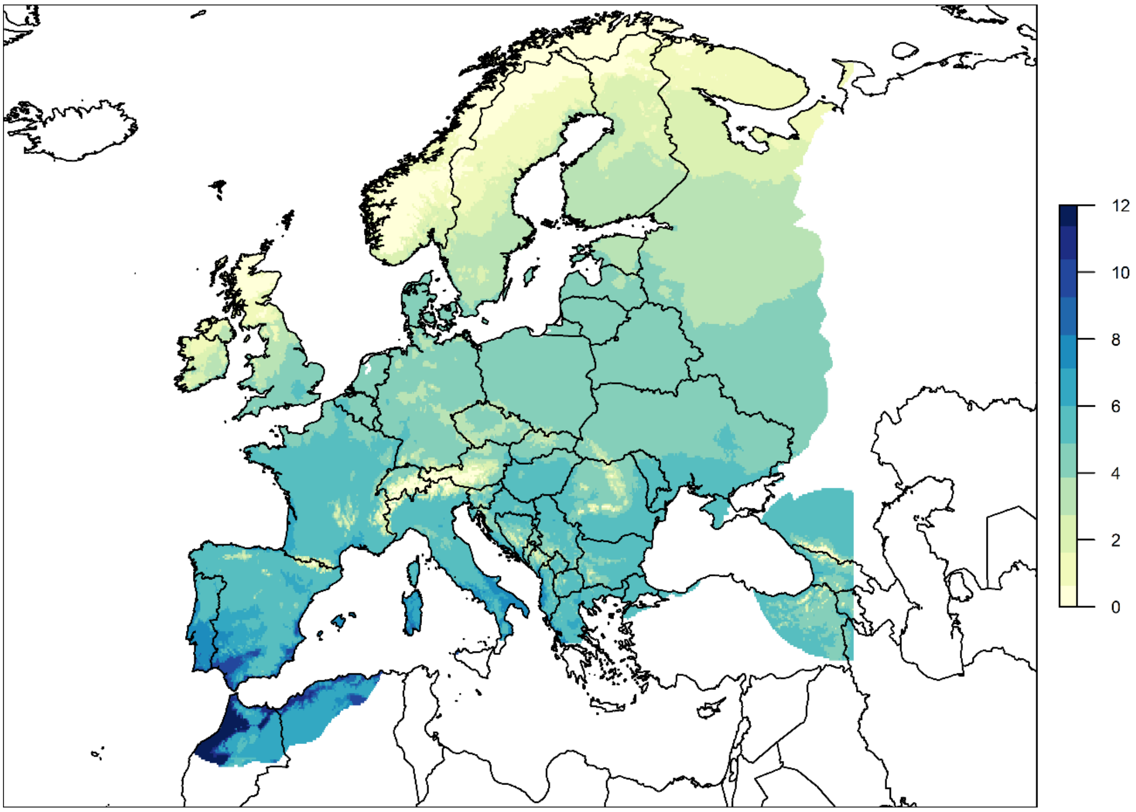

Figure S3: *The number of months in 2020 that demonstrated a permissive suitability for WNV establishment ($S(T) > 0$) at a 1km² cell level across Europe*

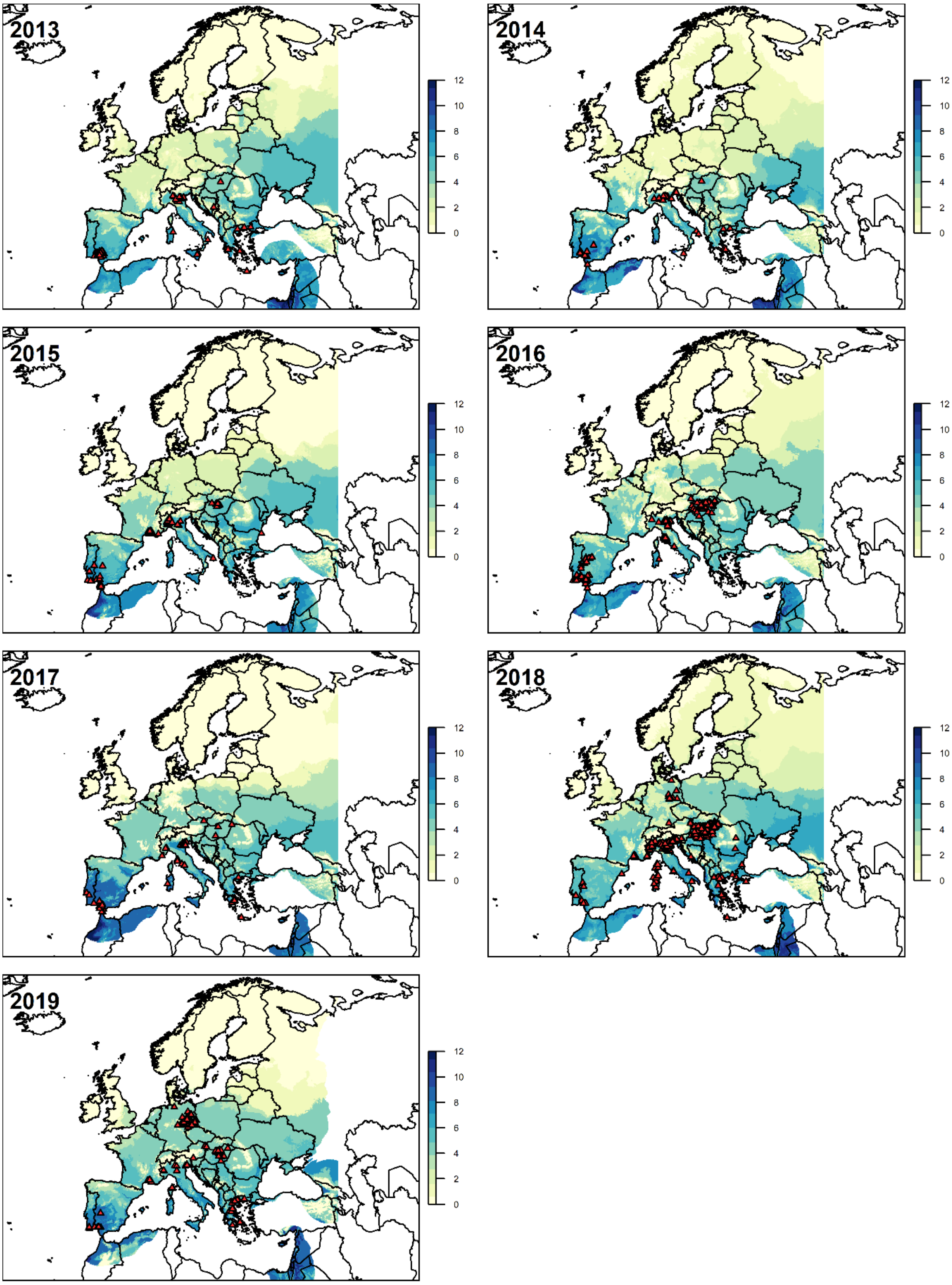

Figure S4: *The number of months that demonstrated a higher suitability for WNV ($S(T) \geq 0.5$) in 2013 to 2019 at a 1km^2 cell level across Europe. Equine cases reported in each year are plotted on top with red triangles.*

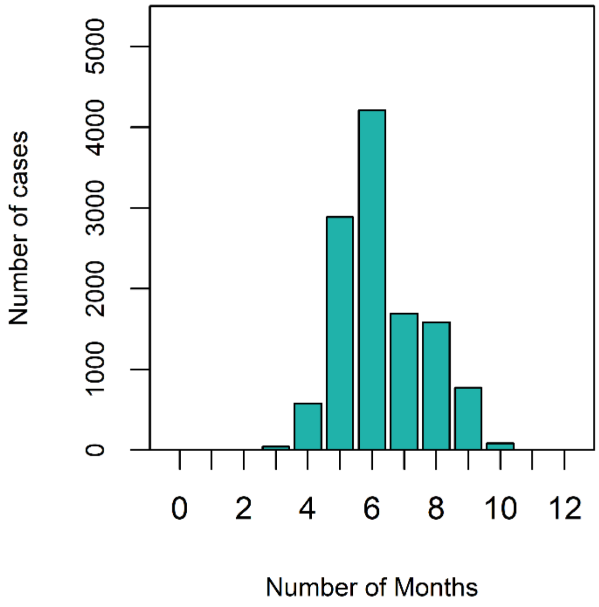

Figure S5: *The number of equine cases from 2013-2019 that occurred in areas with 0-12 months of permissive suitability ($S(T) > 0$). Equine cases (N = 12,771) were extracted from ADNS (ADNS, 2020).*

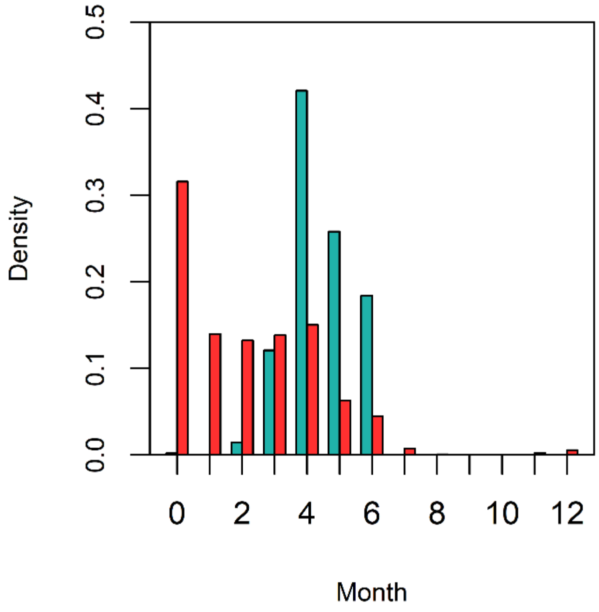

Figure S6*: The density of cells across Europe with different months of higher suitability (red) and the density of cells across Europe with cases with different months of high suitability (green). For all equine cases extracted from ADNS in 2013-2019, and with higher suitability ($S(T) \geq 0.5$) calculated as an average across the years from 2013 to 2019.*

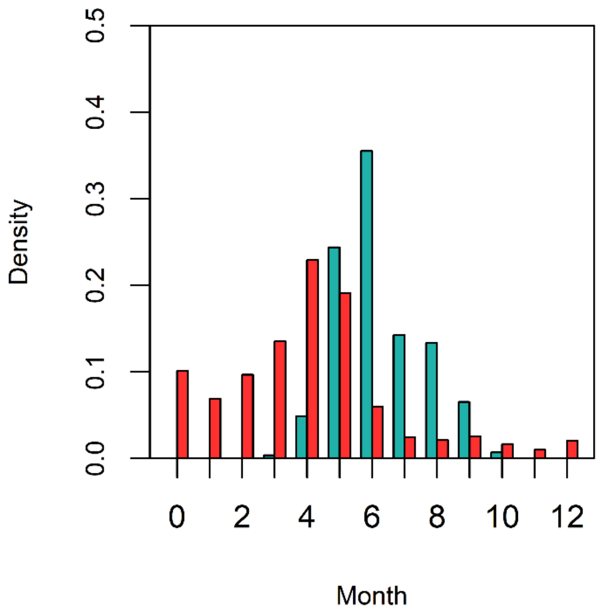

Figure S7: *The density of cells across Europe with different months of permissive suitability (red) and the density of cells across Europe with cases with different months of permissive suitability (green). For all equine cases extracted from ADNS in 2013-2019, and with permissive suitability* ($S(T) \geq 0$) *calculated as an average across the years from 2013 to 2019.*

3. References

ADNS, A. D. N. S. 2020. West Nile Virus ADNS Reports 2013-2020. *In:* COMMISSION, E. (ed.).

ANDREADIS, S., DIMOTSIOU, O. & SAVOPOULOU-SOULTANI, M. 2014. Variation in adult longevity of *Culex pipiens f. pipiens*, vector of the West Nile Virus. *Parasitology research,* 113**,** 4315-4319.

CIOTA, A. T., MATACCHIERO, A. C., KILPATRICK, A. M. & KRAMER, L. D. 2014. The effect of temperature on life history traits of *Culex* mosquitoes. *Journal of medical entomology,* 51**,** 55-62.

DIEKMANN, O. & HEESTERBEEK, J. A. P. 2000. *Mathematical epidemiology of infectious diseases: model building, analysis and interpretation.,* Chichester, Wiley.

DOHM, D. J., O'GUINN, M. L. & TURELL, M. J. 2002. Effect of Environmental Temperature on the Ability of *Culex pipiens* (Diptera: Culicidae) to Transmit West Nile Virus. *Journal of Medical Entomology,* 39**,** 221-225.

FRITZ, M. L., WALKER, E. D., MILLER, J. R., SEVERSON, D. W. & DWORKIN, I. 2015. Divergent host preferences of above- and below-ground *Culex pipiens* mosquitoes and their hybrid offspring. *Med Vet Entomol,* 29**,** 115-23.

HAMER, G. L., KITRON, U. D., GOLDBERG, T. L., BRAWN, J. D., LOSS, S. R., RUIZ, M. O., HAYES, D. B. & WALKER, E. D. 2009. Host Selection by *Culex pipiens* Mosquitoes and West Nile Virus Amplification. *The American Journal of Tropical Medicine and Hygiene,* 80**,** 268-278.

KILPATRICK, A. M., MEOLA, M. A., MOUDY, R. M. & KRAMER, L. D. 2008. Temperature, Viral Genetics, and the Transmission of West Nile Virus by *Culex pipiens* Mosquitoes. *PLOS Pathogens,* 4**,** e1000092.

KOMAR, N., LANGEVIN, S., HINTEN, S., NEMETH, N., EDWARDS, E., HETTLER, D., DAVIS, B., BOWEN, R. & BUNNING, M. 2003. Experimental infection of North American birds with the New York 1999 strain of West Nile virus. *Emerging infectious diseases,* 9**,** 311-322.

LI, J., ZHU, G., ZHOU, H., TANG, J. & CAO, J. 2017. Effect of temperature on the development of *Culex pipiens pallens*. *Chin J Vector Biol & Control,* 28**,** 35-37.

LIM, S. M., BRAULT, A. C., VAN AMERONGEN, G., BOSCO-LAUTH, A. M., ROMO, H., SEWBALAKSING, V. D., BOWEN, R. A., OSTERHAUS, A. D. M. E., KORAKA, P. & MARTINA, B. E. E. 2015. Susceptibility of Carrion Crows to Experimental Infection with Lineage 1 and 2 West Nile Viruses. *Emerging infectious diseases,* 21**,** 1357-1365.

LOETTI, V., SCHWEIGMANN, N. & BURRONI, N. 2011. Development rates, larval survivorship and wing length of *Culex pipiens* (Diptera: Culicidae) at constant temperatures. *Journal of Natural History,* 45**,** 2203-2213.

MADDER, D. J., SURGEONER, G. A. & HELSON, B. V. 1983. Number of Generations, Egg Production, and Developmental Time of *Culex Pipiens* and *Culex Restuans* (Diptera: Culicidae) in Southern Ontario1. *Journal of Medical Entomology,* 20**,** 275-287.

OSÓRIO, H. C., ZÉ-ZÉ, L., AMARO, F., NUNES, A. & ALVES, M. J. 2014. Sympatric occurrence of Culex pipiens (Diptera, Culicidae) biotypes *pipiens, molestus* and their hybrids in Portugal, Western Europe: feeding patterns and habitat determinants. *Med Vet Entomol,* 28**,** 103-9.

RUYBAL, J. E., KRAMER, L. D. & KILPATRICK, A. M. 2016. Geographic variation in the response of *Culex pipiens* life history traits to temperature. *Parasites & Vectors,* 9**,** 116.

SHOCKET, M. S., VERWILLOW, A. B., NUMAZU, M. G., SLAMANI, H., COHEN, J. M., EL MOUSTAID, F., ROHR, J., JOHNSON, L. R. & MORDECAI, E. A. 2020. Transmission of West Nile and other temperate mosquito-borne viruses peaks at intermediate environmental temperatures. *Elife,* 9.

SMITH, D. L., BATTLE, K. E., HAY, S. I., BARKER, C. M., SCOTT, T. W. & MCKENZIE, F. E. 2012. Ross, Macdonald, and a Theory for the Dynamics and Control of Mosquito-Transmitted Pathogens. *PLOS Pathogens,* 8**,** e1002588.

SPANOUDIS, C. G., ANDREADIS, S. S., TSAKNIS, N. K., PETROU, A. P., GKEKA, C. D. & SAVOPOULOU–SOULTANI, M. 2018. Effect of Temperature on Biological Parameters of the West Nile Virus

Vector *Culex pipiens* form '*molestus*' (Diptera: Culicidae) in Greece: Constant vs Fluctuating Temperatures. *Journal of Medical Entomology,* 56**,** 641-650.
VOGELS, C. B. F., HARTEMINK, N. & KOENRAADT, C. J. M. 2017. Modelling West Nile virus transmission risk in Europe: effect of temperature and mosquito biotypes on the basic reproduction number. *Scientific Reports,* 7**,** 5022.
